# Draft Genome Sequence of Teicoplanin-Producing Strain Actinoplanes teichomyceticus CPCC 203265

**DOI:** 10.1128/MRA.01091-19

**Published:** 2019-10-10

**Authors:** Xingxing Li, Cong Zhang, Xuan Lei, Lifei Wang, Bin Hong

**Affiliations:** a NHC Key Laboratory of Biotechnology of Antibiotics, Institute of Medicinal Biotechnology, Chinese Academy of Medical Sciences & Peking Union Medical College, Beijing, China; b CAMS Key Laboratory of Synthetic Biology for Drug Innovation, Institute of Medicinal Biotechnology, Chinese Academy of Medical Sciences & Peking Union Medical College, Beijing, China; Broad Institute

## Abstract

We report here the draft genome sequence of Actinoplanes teichomyceticus CPCC 203265, a producer of glycopeptide antibiotic teicoplanin, which has signiﬁcant inhibitory activity against multidrug-resistant Gram-positive pathogens. The draft genome size is 8 Mb, with a G+C content of 72.8%, and its sequence will facilitate the genome exploration of novel secondary metabolites.

## ANNOUNCEMENT

Actinoplanes teichomyceticus CPCC 203265 (deposited at the China Pharmaceutical Culture Collection [CPCC], original number E92-70) is a Gram-positive bacterium that was isolated from a soil sample from China (1, 2). It produces a glycopeptide antibiotic, teicoplanin, which has signiﬁcant inhibitory activity against multidrug-resistant Gram-positive bacterial pathogens (3). Two almost identical teicoplanin biosynthetic gene clusters have been characterized in A. teichomyceticus strains ATCC 31131 (4) and ATCC 31121 (= DSM 43866 = NRRL B-16726) (5), respectively. Here, we report a novel teicoplanin producer and its draft genome sequence.

For DNA extraction, *A. teichomyceticus* CPCC 203265 was grown for 1 week on potato dextrose agar (PDA; 20% potato, 2% dextrose, and 2% agar) at 28°C from the −80°C glycerol stocks and then transferred to tryptic soy broth (TSB; 2% tryptone, 0.5% NaCl, 0.25% glucose, and 0.25% K_2_HPO_4_ [pH 7.2]) at 28°C for 48 h. The genomic DNA was isolated using a DNA extraction kit (TANBead, China) and assessed using a NanoDrop ND-8000 spectrophotometer and agarose electrophoresis. Genomic DNA libraries were prepared using the NEBNext Ultra DNA library prep kit for Illumina (New England BioLabs, UK) and then sequenced on an Illumina HiSeq 2000 platform at the Beijing Genomics Institute (BGI; Shenzhen, China). A total of 1,119 Mb of paired-end raw reads (2 × 90 bp) were generated. After sequencing, adapters were trimmed and low-quality reads were filtered out using readfq v5 with default settings (http://github.com/cjfields/readfq). A total of 841 Mb of clean data (9,344,446 reads with a 461-bp average insert size and 100-fold average coverage) was obtained. The draft genome sequence was directly assembled into 102 contigs (8,226,041 bp, *N*_50_ of 210,764 bp, and G+C content of 72.8%) and then composed into 56 scaffolds (8,226,652 bp, *N*_50_ of 417,241 bp, and G+C content of 72.8%) by SOAPdenovo v2.04 (parameter settings, -K 63 -F -M -d 1 -R -u) (6).

Genome mining analysis using antiSMASH 5.0 with default settings (https://antismash.secondarymetabolites.org/) (7) revealed the presence of 28 putative gene clusters responsible for the production of diverse secondary metabolites in the draft genome sequence of *A. teichomyceticus* CPCC 203265. Of these, the putative teicoplanin biosynthetic gene cluster was located on scaffold 23, and its gene composition shows a high similarity with the reported teicoplanin clusters (4, 5). This suggested that the teicoplanin clusters might be highly conserved in *A. teichomyceticus.* Genome mining analysis by antiSMASH also revealed some gene clusters related to the biosynthesis of analogues of teichomycin, desferrioxamine, sioxanthin, and micromonolactam, as well as a number of clusters which are not highly conserved with any known cluster. The genome sequence of *A. teichomyceticus* CPCC 203265 provides insights into its metabolic potential and will help in future valuable applications.

### Data availability.

This draft genome sequence of A. teichomyceticus CPCC 203265 has been deposited at DDBJ/ENA/GenBank under the BioProject number PRJNA562825, BioSample number SAMN12651337, and accession number VTPA00000000. The version described in this paper is the first version, VTPA01000000. Clean sequence reads have been deposited in the Sequence Read Archive (SRA) database under the accession number SRR10034702. *A. teichomyceticus* CPCC 203265 is deposited at the China Pharmaceutical Culture Collection.
